# Supplementary material for: Kinetic Changes in B7 Costimulatory Molecules and IRF4 Expression in Human Dendritic Cells during LPS Exposure
Source: Biomolecules. 2022 Jul 8;12(7):955. doi: 10.3390/biom12070955 (PMC9313461; doi:10.3390/biom12070955)
Supplement: Supplementary file 1 [file biomolecules-12-00955-s001.zip › biomolecules-1788251-supplementary.pdf]

Supplementary Material

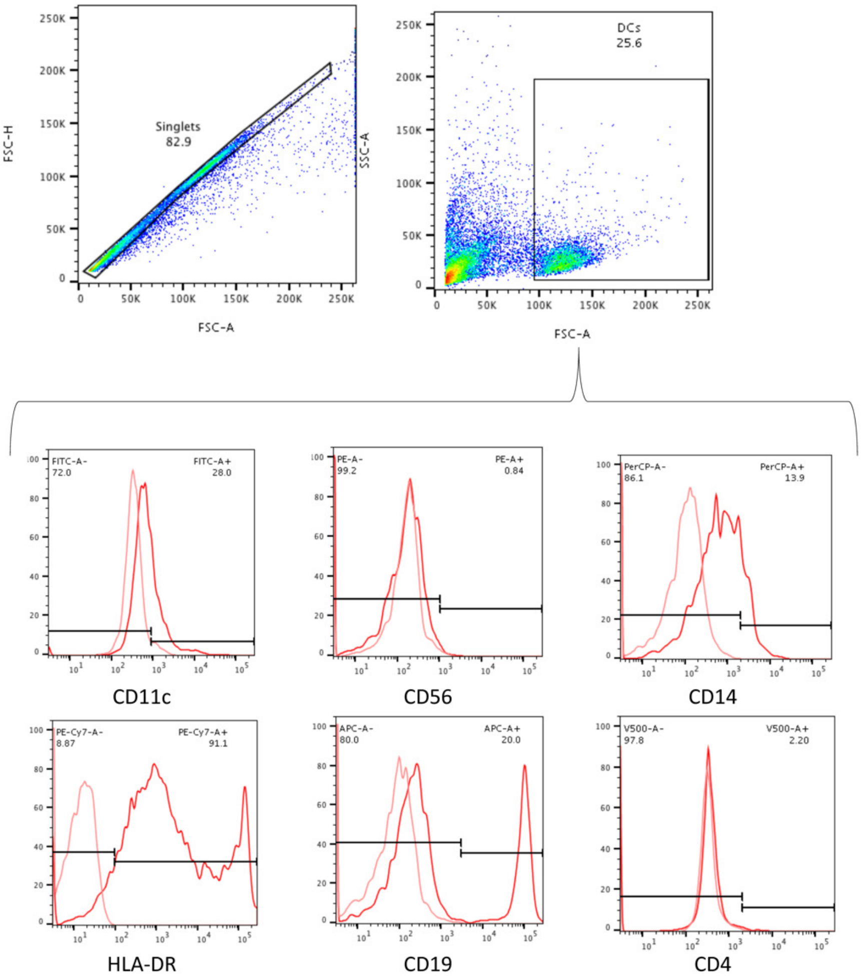

CD	MFI	Percentage
HLA-DR	21752	91.1
CD14	970	13.9
CD11c	1478	28
CD4	683	2.2
CD19	18377	20
CD56	160	0.84

Stained
Unstained

Supplementary Figure S1. Mo-DCs immunophenotype. After 7 days of culture with recombinant GM-CSF and IL-4, differentiated Mo-DCs immunophenotype was evaluated for the HLA-DR, CD14, CD11c, CD4, CD19, and CD56 antigen.

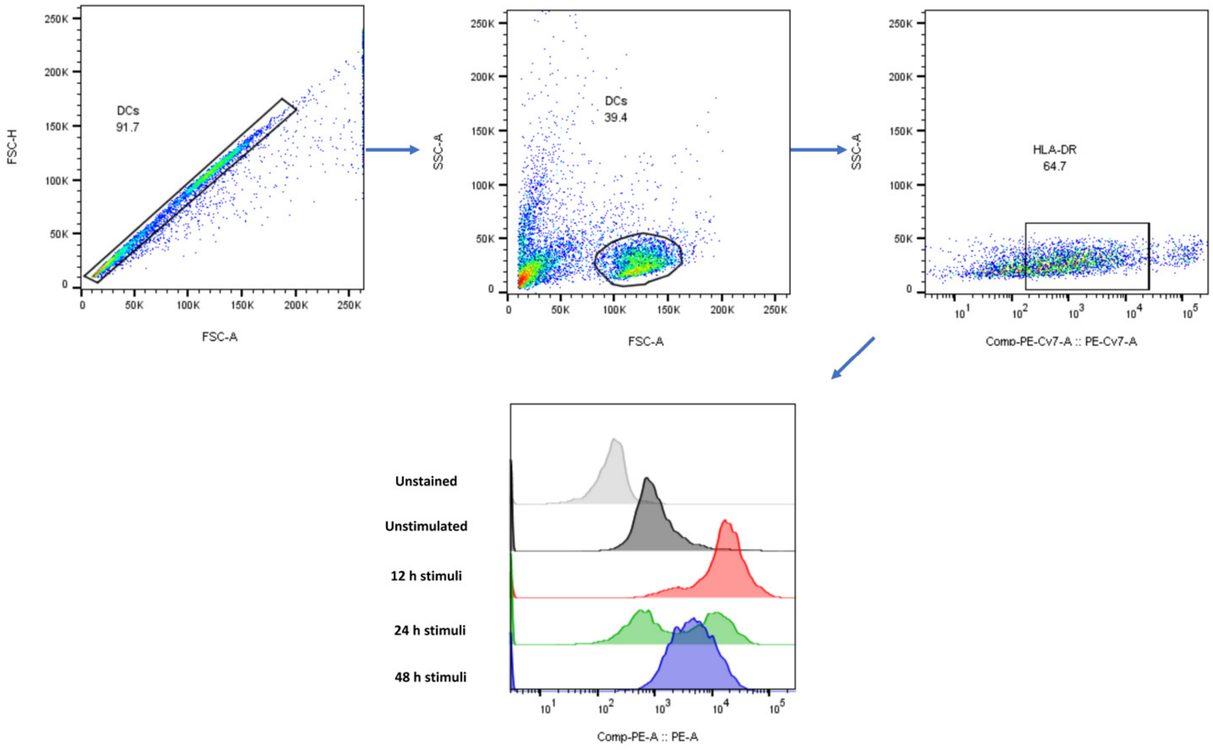

Supplementary Figure S2. Flow cytometry analysis strategy. The analysis consisted of gating Single Cells (FSC-H vs. FSC-A), then size and complexity events suggestive of Mo-DCs were gated (FSC-A vs. SSC-A), and HLA-DR + cells were considered Mo-DCs.

Supplementary Table S1. Kinetic of B7 Costimulatory molecules and IRF-4 expression after *LPS* stimulation of Mo-DCs cells

B7 Molecule	Time of stimulation				Statistical significance
	US	12h	24h	48h	*p*
**CD86 100ng**	437 (242-1123)*	399 (236.5-1135)	516 (340-1193)	951 (558-5623)	*0.021
**CD86 1 ug**	437 (242-1123)*	502 (264.5-1292)	695 (479-1103)	688 (620-5277)*	*0.044
**CD86 10ug**	437 (242-1123)*	387 (243-1271)	707 (356-1636)	680 (560-3474) *	*0.044
**ICOSL 100ng**	526 (272-1135)*	1259 (952-1755)	925 (542-3280)	1395 (891.5-2576)*	*0.0066
**ICOSL 1ug**	526 (272-1135)*	1684 (981-2538)*	907 (505-3743)	1062(842-221)	*0.0034
**ICOSL 10ug**	526 (272-1135)*	1462 (807-2089)	759(442-3728)	1768 (940-2850)*	*0.0066
**PDL1 100ng**	1523(1217-2483)*¶¥	4132(1681-4554)*	3389 (3181-4851)¶	3426(2987-8315)¥	*0.012,¶<0.0001,¥0.0003
**PDL1 1ug**	1523(1217-2483)*¶¥	33548 (2487-5079)*	5073(3496-6193)¶	5511(3933-9136)¥	*0.0003,¶<0.0001,¥<0.001
**PDL1 10ng**	1523(1217-2483)*¶¥	2973 (1863-3465)*	3805(3634-5782)¶	4293(3824-9020)¥	*0.003,¶<0.0001,¥<0.0001
**PDL2 100ng**	1238(725-1501)*	1508 (1268-2692)*	1004(295-2212)	1474 (1321-1706)	*0.0170
**PDL2 1 ug**	1238(725-1501)*¶	1555(1281-3835)*	1055(896-1759)	1789(1619-1904)¶	*0.0259,¶0.0009
**PDL2 10ug**	1238(725-1501)*¶	1655 (1348-3801)*	1205(1074-1797)	1609(1397-2107)¶	*0.0096,¶0.0199

US.- Unstimulated. Mean fluorescence intensity for CD86, ICOS-L, PDL1, and PDL2 on Mo-DCs. MoDCs from healthy donors were stimulated with LPS for 12, 24, or 48 h with 100ng, 1 μg, or 10 μg of LPS or left unstimulated (US). The Median and IQR of 3 independent determinations for every costimulatory molecule performed by triplicate are depicted.

Supplementary Table S2. Kinetic of soluble cytokines after *LPS* stimulation of Mo-DCs cells

Cytokine	Time of stimulation				Statistical significance
	US	12h	24h	48h	*p*
**TNF-α 100 ng**	1.93±0.6437*¶	812.2±278.7*¥	547.1±96.17¶	213.1±24.2¥	*0.007,¶0.0085,¥0.0049
**TNF-α 1μg**	1.93±0.6437*¶	1406±51.6*¥	778.1±262.8¶Φ	290±66.37¥Φ	*<0.0001,¶0.0006,¥<0.0001,Φ0.0107
**TNF-α10μg**	1.93±0.6437*¶¥	1182±75.75*¶Φ	923.8±145	354.1±98.75Φ	*<0.0001,¶<0.0001,¥<0.0085,Φ<0.0001
**IL-6 100 ng**	0.4833±0.4252*¶¥	1589±389.5*	2122±491.6¶	2038±289.3¥	*0.0022,¶0.003,¥0.004
**IL-6 1μg**	0.4833±0.4252*¶¥	3468±684.3*	2375±1095¶	2369±484.3¥	*0.0012,¶0.0125,¥0.127
**IL-6 10μg**	0.4833±0.4252*¶¥	2868±785.6*	2495±693.7¶	3194±907.4¥	*0.0043,¶0.0097,¥0.0022
**IL-10 100 ng**	0.2067±0.358*	15.31±6.757	23.28±9.245*	15.38±3.913	*0.007
**IL-10 1μg**	0.2067±0.358*	48±9.982*	26.62±21.29	15.3±8.726	*0.007
**IL-10 10μg**	0.2067±0.358*	35.06±19.29*	21.76±7.466	24.89±10.34	*0.0253
**IFN-γ 100 ng**	0±0*	10.91±3.627¶	35.64±27.42¥	140.7±59.75*¶¥	*0.0047,¶0.0065,¥0.0178
**IFN-γ 1μg**	0±0*	21.17±4.69¶	97.47±39.96¥	572.4±133.5*¶¥	*<0.0001,¶<0.0001,¥0.0001
**IFN-γ 10μg**	0±0*	21.11±16.57¶	195±46.82¥	957.8±413*¶¥	*0.0022,¶0.0025,¥0.0087
**IL-2 100 ng**	0±0	0.2033±0.3522	0.6967±0.2892	3.817±3.816	
**IL-2 1μg**	0±0*	1.127±0.9981	1.64±0.6235	2.69±0.7712*	*0.0068
**IL-2 10μg**	0±0*¶	1.653±0.774¥	3.143±0.7006*	4.323±1.43¶¥	*0.0105,¶0.0015,¥0.0252

US.- Unstimulated. The supernatant was collected and assessed for the soluble cytokines TNF-α, IL-6, IL-10, INF-y, and IL-2 as described in the material and methods. The mean ± SD of 3 independent assays performed by triplicate is depicted.

Supplementary Table S3. Concentration of soluble CD86, ICOS-L, PDL-1, and PDL2 in supernatant of Mo-DCs cells after *LPS* stimulation

LPS concentration	Soluble costimulatory molecule ng/mL		
**12h**	**sCD86**	**sPDL-1**	**sPDL-2**
US	203±15.41*	419.8±6.249*	399.2±29.17*
100 ng	166.5±31.68*	441.7±12.5*¶	360.4±7.765*¶
1μg	167.6±21.5*	455.3±32.47*¶	324.5±24.96*¶
10μg	158.2±21.6*	464.8±70.43*	405.2±16.16*
**24h**	**sCD86**	**sPDL-1**	**sPDL-2**
US	203±15.41*	419.8±6.249*	399.2±29.17*
100 ng	158.2±17.44*	448.5±4.724*¶	364.8±15.53*¶
1μg	183±5.4*	376.2±43.74*	346.9±28*
10μg	178.2±21.5*	445.7±45.06*¶	342.4±42.76*¶
**48h**	**sCD86**	**sPDL-1**	**sPDL-2**
US	203±15.41*	419.8±6.249*	399.2±29.17*
100 ng	158.2±17.44*	448.5±4.724*	364.8±15.53*
1μg	183±5.4*	376.2±43.74*¶	346.9±28*¶
10μg	178.2±21.5*	445.7±45.06*	342.4±42.76*

US.- Unstimulated. MoDCs from healthy donors were stimulated with LPS for 12, 24, or 48 h with 100ng, 1 μg, or 10 μg of LPS or left unstimulated (US). The supernatant was collected and assessed for soluble costimulatory molecules as described in the material and methods. (ICOS-L was not detected in any of the conditions evaluated). The mean ± SD of 3 independent assays performed by triplicate is depicted. * p<0.001 ¶ p<0.05

Supplementary Table S4. Correlation matrix Cytokines *vs.* costimulatory molecules and IRF4.

		Costimulatory Molecules				
		**CD86**	**ICOSL**	**PDL1**	**PDL2**	**IRF-4**
**Cytokines**	TNF-α	-0.54	0.25	-0.19	0.65	-0.31
	IL-6	0.31	0.32	0.32	0.45	-0.18
	IL-10	0.04	0.54	0.07	0.41	-0.45
	IFN-γ	0.89*	0.47	0.93*	-0.07	0.07
	IL-2	0.86*	0.35	0.67*	0.01	0.03

r value for the Spearman correlation test is depicted. *p<0.05
